# Local effect of allopregnanolone in rat ovarian steroidogenesis, follicular and corpora lutea development

**DOI:** 10.1038/s41598-024-57102-1

**Published:** 2024-03-16

**Authors:** Antonella Rosario Ramona Cáceres, Daniela Alejandra Cardone, María de los Ángeles Sanhueza, Ignacio Manuel Bosch, Fernando Darío Cuello-Carrión, Graciela Beatriz Rodriguez, Leopoldina Scotti, Fernanda Parborell, Julia Halperin, Myriam Raquel Laconi

**Affiliations:** 1 Laboratorio de Fisiopatología Ovárica, Instituto de Medicina y Biología Experimental de Cuyo (IMBECU – CONICET Mendoza), Av. Ruiz Leal s/n Parque General San Martín, CP 5500 Mendoza, Argentina; 2 Facultad de Ingeniería y Facultad de Ciencias Médicas, Universidad de Mendoza, Mendoza, Argentina; 3 C&S Argentina, I+D+I, Buenos Aires, Argentina; 4 Laboratorio de Oncología, Instituto de Medicina y Biología Experimental de Cuyo (IMBECU – CONICET Mendoza), Mendoza, Argentina; 5 Facultad de Química, Bioquímica y Farmacia, Universidad Nacional de San Luis, San Luis, Argentina; 6 Ovarian Pathophysiology Studies Laboratory, Institute of Experimental Biology and Medicine (IByME) - CONICET, Buenos Aires, Argentina; 7 Centro de Estudios Biomédicos Básicos, Aplicados y Desarrollo (CEBBAD), Universidad Maimónides, Ciudad Autónoma de Buenos Aires, Argentina

**Keywords:** Microscopy, Gene expression analysis, Reverse transcription polymerase chain reaction, Gonads

## Abstract

Allopregnanolone (ALLO) is a known neurosteroid and a progesterone metabolite synthesized in the ovary, CNS, PNS, adrenals and placenta. Its role in the neuroendocrine control of ovarian physiology has been studied, but its in situ ovarian effects are still largely unknown. The aims of this work were to characterize the effects of intrabursal ALLO administration on different ovarian parameters, and the probable mechanism of action. ALLO administration increased serum progesterone concentration and ovarian 3β-HSD2 while decreasing 20α-HSD mRNA expression. ALLO increased the number of atretic follicles and the number of positive TUNEL granulosa and theca cells, while decreasing positive PCNA immunostaining. On the other hand, there was an increase in *corpora lutea* diameter and PCNA immunostaining, whereas the count of TUNEL-positive luteal cells decreased. Ovarian angiogenesis and the immunohistochemical expression of GABA_A_ receptor increased after ALLO treatment. To evaluate if the ovarian GABA_A_ receptor was involved in these effects, we conducted a functional experiment with a specific antagonist, bicuculline. The administration of bicuculline restored the number of atretic follicles and the diameter of *corpora lutea* to normal values. These results show the actions of ALLO on the ovarian physiology of the female rat during the follicular phase, some of them through the GABA_A_ receptor. Intrabursal ALLO administration alters several processes of the ovarian morpho-physiology of the female rat, related to fertility and oocyte quality.

## Introduction

Allopregnanolone (ALLO; IUPAC: 1-[(3R,5S,8R,9S,10S,13S,14S,17S)-3-hydroxy-10,13-dimethyl-2,3,4,5,6,7,8,9,11,12,14,15,16,17-tetradecahydro-1H-cyclopenta[a]phenanthren-17-yl]ethenone) is an endogenous steroid synthesized de novo or by progesterone (P4) metabolism in the nervous system, adrenal glands, ovaries and placenta in the female^1–5^. ALLO is mostly known for its anxiolytic, antidepressant, anticonvulsant and neuroprotective actions at the central nervous system (CNS) level^6–9^. Moreover, it can modulate a variety of behaviors, such as reproductive^10–13^, social, exploratory and affective conducts^14–16^. Previous work from our laboratory reported ALLO actions over the hypothalamic–pituitary–gonadal axis when administered via ICV^17–19^ and its effects on the ovarian peripheral innervation using an ex vivo culture of the superior mesenteric ganglion- ovarian nervous plexus—ovarian system^20–22^. These findings led us to consider ALLO not only as a modulator of central function but also of ovarian physiology.

The ovary is one of the major sources of circulating ALLO^23,24^, and the ovarian cells express many membrane receptors that could be action sites for its in situ reproductive modulation. ALLO is synthesized by ovarian interstitial cells, follicles and *corpora lutea* (CL)^25^. Its serum concentrations vary along the rat estrous cycle^24^, human menstrual cycle and gestation^26^, following P4 levels^24,27^, and its ovarian synthesis is stimulated by LH^28^. The changes in steroid hormones serum concentration is also accompanied by an increase in their ovarian concentration^24^. However, the direct actions of ALLO over the ovarian physiology are not known.

ALLO is considered a potent positive allosteric modulator of GABA_A_ receptors (GABA_A_R), enhancing GABAergic activity at nanomolar concentrations and directly activating the receptor at micromolar concentrations^29–33^. There are few reports of the presence and function of GABA_A_R in non-neural tissue^34^. The presence of GABA_A_R, GABA and the enzyme glutamate acid decarboxylase (GAD) has been previously demonstrated in the ovary^34–36^. Ovarian GABA concentrations change throughout the estrous cycle^37^ and gestation^38,39^. An example of non-neural GABAergic action occurs in the uterus of pregnant rats, where ALLO exerts its effects by regulating the contractions of the rat’s myometrium^40–42^. In addition, in pseudopregnant rats, an intrabursal perfusion of GABA (0.5 µM) produces vasoconstriction, a decrease in P4 secretion, and an immediate increase in estrogen secretion^43^. The importance of P4 metabolite-mediated regulation of non-neural GABA_A_R present in the rat reproductive system remains to be fully elucidated.

To evaluate the action of a GABA_A_R modulator, it is important to test its functional activity by using an antagonist. Briefly, in a receptor functional study, the antagonist prevents the action of the agonist, thus avoiding its effect. This confirms that the receptor is indeed involved in the agonist’s actions. Bicuculline (BIC) is a benzylisoquinoline alkaloid and a competitive antagonist of GABA_A_R^44^. In a primary culture of astrocytes, the half-maximal inhibitory concentration (IC50) of bicuculline on GABA_A_Rs is 3.3 μM for a concentration of 30 μM GABA. It shows a Hills coefficient of − 1.8 (± 0.06), indicating negative cooperativity at the receptor (initial binding inhibits subsequent binding to other receptor sites)^45^. Bicuculline sensitivity is defined by IUPHAR as an important criterion in the definition of GABA_A_Rs^46–48^, and it is the most frequently used antagonist for GABA_A_R functional studies.

Bearing in mind the previous findings, we analyzed ALLO effects on the local regulation of ovarian key features using an in vivo intrabursal administration model. We evaluated (a) changes in ovarian morphometry, weight and ovulation, (b) ovarian angiogenesis, (c) apoptosis and proliferation of granulosa and luteal cells, d) steroidogenesis and hormone secretion. Finally, in order to determine whether the actions of ALLO involve this receptor, we performed a GABA_A_R functional experiment with bicuculline. This is the first evidence of the local effect of ALLO on the female rat ovarian function and its eventual pathological consequences.

## Materials and methods

### Reagents

Allopregnanolone (C_21_H_34_O_2_, 3α-hydroxy-5α-pregnan-20-one, Sigma Chemical Co., St. Louis, MO, USA, CAS Number 516-54-1), Ketamine HCl (Ketonal 50 mg/ml, Richmond Laboratories, Veterinary Division, Buenos Aires, Argentina) and Xylazine (Sedomin 100 mg/ml, König Laboratories, Buenos Aires, Argentina) were used for experimental and surgical procedures.

To obtain the stock solution, ALLO was first dissolved in propylene glycol at a concentration of 3.14 mM. Working concentration was obtained after serial dilutions in sterile physiological solution; the resulting propylene glycol concentration was less than 0.2%.

Bicuculline methiodide [1(S),9(R)-(-)-Bicuculline methiodide, Sigma, USA] was used to antagonize GABA_A_Rs. A stock solution was prepared in DMSO at an initial concentration of 1 × 10^−2^ M. Working concentrations were obtained by dilution of the initial one in physiological solution; the resulting DMSO concentration at working solutions was less than 0.2%.

The control group received physiological solution (BRAUN, Buenos Aires, Argentina) with a similar concentration of propylene glycol/DMSO.

Bouin solution (Biopur Diagnostics, Santa Fe, Argentina), hematoxylin, eosin (Merk, Germany) and Canada Balsam Synthetic (Biopack, Buenos Aires, Argentina) were used for histological procedures.

### Animals

Adult female Sprague Dawley rats (*Rattus norvegicus*) of 200–250 g were maintained under controlled conditions of temperature and light (12 h light/12 h darkness photoperiod). They were housed in groups of four animals *per* cage, with food (standard rat chow, Cargill, Córdoba, Argentina) and water available ad libitum. Vaginal smears from each rat were observed daily (07:00–09:00 am) with a light microscope (Zeiss, Germany) to determine the stage of the estrous cycle. Only those animals exhibiting two or more consecutive 4 or 5-day cycles were used. All protocols were previously approved by the Institutional Committee for the Care and Use of Laboratory Animals of the National University of Cuyo (CICUAL aval: 154/2019). The animals for these experiments were kept and handled according to the National Institutes of Health Guide for the care and use of laboratory animals, 8th edition (National Research Council). The study was performed, and results reported in accordance to ARRIVE guidelines. The total number of animals used for the experiments was 35.

### Anesthesia

A combination of ketamine (80 mg/kg) and xylazine (10 mg/kg) administered intraperitoneally was used. Confirmation of the level of anesthesia was assessed by loss of postural reflex, palpebro-palpebral reflex, corneo-palpebral reflex, podal reflex and lack of response to tail pinch.

### Surgical procedure

Access to each ovary was made through two paramedian ventral incisions. This procedure allowed gentle handling of the tissue and correct visualization of the inoculation site, and thus to evaluate the complete discharge of the inoculum and the integrity of the post-manipulation bursa.

**Figure 1:**
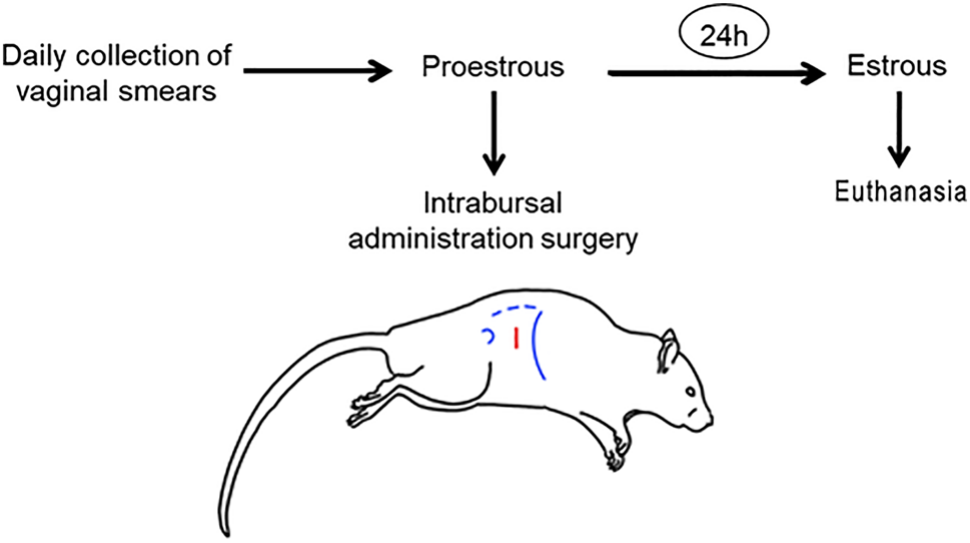
Experimental design. On the day of proestrus the rats were subjected to an intrabursal administration surgery. On the day of estrous, 24 h later, they were euthanized by decapitation. Blue lines show the anatomical references used for the surgery: the greater trochanter of the femur, the vertebral column and the last rib. The red line shows the area of incision.

With the animal in lateral decubitus, a skin incision was made no more than 10 mm caudal to the last rib (Fig. 1), taking as anatomical references the greater trochanter of the femur and the vertebral column. The ovary was carefully lifted with forceps, taking it by one of the fat pads that surround it. Once outside the abdominal cavity, the ovary was placed on a pad of wet gauze adjacent to the incision site to avoid dehydration of the structures. The corresponding treatment (5 µl of total volume) was then injected using a Hamilton type syringe. Correct inoculation was corroborated by visualizing the needle and dilation of the post-injection bursa. The needle was quickly withdrawn with care not to tear the ovarian bursa. Then, the ovary was carefully reintroduced into the abdominal cavity and each of the incised planes was sutured. The described procedure was performed on each abdominal side, taking less than 8 min in total. After surgery, the animals were kept under observation in a heated room until they fully recovered from anesthesia. After 24 h, the animals were in perfect condition with no signs of pain or lethargy.

The ovarian intrabursal drug delivery model was used to analyze the local effects of ALLO on ovarian physiology. This model allows in vivo topical administration of drugs to the ovary, keeping the anatomy, innervation, and blood flow intact^49^. Treatments inoculated into the bursal cavity rapidly penetrate the ovarian parenchyma^50,51^. In this way, the ovulation rate and in situ response to the applied drugs can be assessed while the ovary is under systemic regulation.

### Experimental design

On the morning of proestrus, 5 µl of ALLO at a concentration of 6 µM and controls were administered intrabursally (Fig. 1). The ALLO concentration of 6 µM was chosen for these studies based on a concentration–response curve (data not shown) and previous works. It was the pharmacological concentration with the greatest effects on ovarian physiology^19,22^.

The inoculation volume was determined based on the work of Abramovich et al.^52^. On the morning of estrus, 24 h later, the animals were euthanized by guillotine decapitation. Confirmation of estrous was performed prior to euthanasia. The ovaries were removed and placed in glass Petri dishes with chilled saline solution for subsequent assays. Serum samples were collected and stored at − 80 °C until processing. All the counts were recorded by a researcher blinded to the group.

*Experiment 1* A paired model was used to reduce the variability between groups. Thus, the left ovary of each animal received ALLO 6 µM and the right ovary (control) received saline. A paired study is possible since the changes produced by the inoculation of treatments in one ovary are not compensated by the contralateral^49^, at least until 24 h after their injection^53^. Also, the intrabursal administration of saline solution, commonly used in the control ovary, does not alter ovarian physiology^54,55^. For serum hormone determinations, both ovaries of two groups of animals were treated with saline or ALLO 6 µM (not paired experiment). The total number of animals used for this experiment was 15.

*Experiment 2* To evaluate whether some of the ALLO actions on ovarian morphometry were mediated by GABA_A_R, a functional assay was performed with bicuculline, a competitive GABA_A_R antagonist. We tested 3 commonly used doses: 5, 10 and 100 µM^56–58^. None of the BIC concentrations per se induced significant changes in the evaluated parameters (data not shown). Therefore, the 10 µM concentration was selected for this experiment. In this case, both ovaries were injected with the treatments (n = 5 animals/group). For this experiment the total number of animals used was 20.

### Evaluation of ovulation and ovarian weight

After removal of the ovaries, the distended portion of the ampullae was located and punctured with a 21-gauge needle under a stereoscopic magnifying glass (Zeiss, Germany). The number of oocytes released were then counted for each ovary. To clean the ovary, the adhering tissue and fat were dissected out and discarded. The ovaries were weighted on an analytical scale. The number of animals used was 5, with 5 ovaries *per* experimental group.

### Ovarian morphometry

After ovarian weight analysis, the ovaries were immediately fixed in Bouin solution for 12 h. They were then dehydrated in a series of increasing concentrations of ethyl alcohol and finally embedded in paraffin. Paraffin sections of 5 μm were taken every 40 μm and mounted onto microscope slides to prevent counting the same structure twice, according to the method described by Woodruff et al.^53^. Finally, the slides were stained with hematoxylin–eosin and mounted with mounting medium.

Follicles were classified as primary (a single layer of cuboidal granulosa cells), secondary (more than one layer of granulosa cells with an incipient antrum), tertiary (more than three granulosa layers and a clearly defined antral space) or Graafian follicles (polarized oocyte, defined cumulus granulosa layer and a cavity occupying most of the follicular volume). *Corpora lutea* were identified by the presence of large and small luteal cells with their characteristic cytoplasmic eosinophilia^59^. *Corpora albicans* were scarce, and they were not taken into account. Morphological characteristics of atretic follicles (ATF) include pyknotic nuclei (cell degeneration) in more than 5% of the total number of granulosa cells, detachment of the granulosa cell layer from the basal laminae, hypertrophy of external theca and oocyte degeneration^60^. Follicular cysts were identified by a thin granulosa layer (≤ 3 mm) and a large antral cavity (the oocyte may be absent or present)^61^. The number of ovarian structures was determined in four ovarian sections from each ovary (n = 5 animals/group). The structure diameters were measured using Image J software (Image Processing and Analysis in Java; National Institutes of Health, Bethesda, MD, USA). Results were expressed as mean diameter ± SEM.

### In situ cell death detection (TUNEL)

For quantification of apoptosis in follicles and CL, 3 µm thick paraffin sections were processed for in situ localization of nuclei showing DNA fragmentation by the TUNEL technique (terminal-end-labeling with dUTP-digoxigenin using the enzyme deoxynucleotidyl transferase (TdT)). Ovarian sections were deparaffinized and hydrated by washing in ethyl alcohol of decreasing concentration (100°–70°). They were then incubated with Proteinase K 20 μg/ml (Promega, Madison, WI, USA) for 10 min at room temperature, followed by incubation in 0.01 M citrate buffer pH 6 for 30 min at 90–100 °C. The *In-situ Cell Death Detection KIT, POD* (Roche, Mannheim, Germany) was used. The labeling reaction was performed according to the manufacturer’s protocol.

The preparations were incubated with digoxigenin-dUTP and TdT enzyme (labeling solution and enzyme solution) for 1 and 30 min, respectively. They were then incubated with peroxidase-conjugated anti-digoxigenin monoclonal antibody for 30 min in a humid chamber at 37 °C. Finally, apoptotic positive cells were visualized after reaction with diaminobenzidine (SIGMAFAST™ 3,3′-diaminobenzidine tablets, Sigma, Darmstadt, Germany). Negative controls were obtained by replacing the TdT solution with phosphate buffer pH 7. The sections were counterstained with hematoxylin. The number of apoptotic cells was determined by counting labeled cells in follicles in randomly selected fields at 400 × magnification (n = 5 ovaries/group). The apoptotic index was calculated as the percentage of apoptotic cells relative to the total number of cells.

### Immunohistochemistry

Ovarian tissue sections were cut at 3 µm and mounted on positively charged slides. Tissue sections were deparaffinized in xylene and rehydrated by washing with alcohols. Antigen retrieval was performed in 0.01 M sodium citrate buffer at pH 6 for 10 min in a microwave oven at maximum power (800 W).

Endogenous peroxidase enzyme activity was blocked with 3% hydrogen peroxide solution in PBS buffer for 10 min. Blocking of non-specific binding sites was performed in a humidity chamber by incubating the sections with 2% BSA (bovine serum albumin) for 20 min at room temperature. The histological sections were then incubated with 20 µl of the primary antibodies listed in Table 1 overnight at 4 °C in a humidity chamber.

**Table 1:** Antibodies used for immunohistochemistry.

Antibody target	Host/type	Supplier	Cat. N°	Dilution
PCNA	Rabbit Polyclonal	Santa Cruz Biotechnology, USA	sc-7907	1:300
α-smooth muscle actin	Mouse Monoclonal	Santa Cruz Biotechnology, USA	sc-56499	1:100
Von Willebrand factor	Rabbit Polyclonal	Dako Cytomation, USA	A0082	1:100
GABA_A_ receptor Rα1-6	Mouse Monoclonal	Santa Cruz Biotechnology, USA	sc-376282	1:200
ANTI-rabbit HRP IGG	Goat Polyclonal	Sigma Aldrich, USA	A4914	1:1000
ANTI-mouse HRP IGG	Goat Polyclonal	R&D Systems, USA	HAF007	1:1000

A negative control mouse IgG (I-2000-1, Vector Laboratories, Burlingame, CA, USA) was used instead of the primary mouse monoclonal antibody to evaluate non-specific binding. A secondary negative control was performed by omitting incubation of the sections with the primary antibody. The next day, after successive washes with PBS, the sections were incubated with the appropriate secondary antibodies listed in Table 1 for 2 h. They were then incubated with ABC: avidin-biotinylated peroxidase complex (Vectastain ABC system, Vector Laboratories, Burlingame, CA, USA) for 30 min. Positive labeling was visualized by precipitation of the peroxidase enzyme substrate, diaminobenzidine (DAB), in developer buffer (Roche Diagnostics, Germany). The reaction was stopped with distilled water and counterstained with hematoxylin for 1 min. Upon completion of immunolabeling, photographs were taken with a digital camera attached to a light microscope (Nikon, Melville, NY, USA) for further processing. Image J software was used to count positively labeled cells and total cells per structure.

Cell proliferation was evaluated by PCNA (proliferating cell nuclear antigen) in secondary, tertiary and de Graaf follicles as well as in CL. This group of follicles is the most susceptible to degeneration by atresia, since they depend on gonadotrophin stimulation for their survival and can sense the balance between external pro/anti-apoptotic factors^62,63^. The proliferation index was calculated for each field using the following formula: number of immunolabeled cells/total number of cells counted per field. The number of apoptotic cells was determined by counting labeled cells in follicles in randomly selected fields at 400 × magnification (n = 5 ovaries/group). Results were expressed as mean ± SEM of cells positive for the selected antigen.

The percentage of positively labeled endothelial (von Willebrand factor-labeled) and peri-endothelial (α-actin-labeled) areas relative to the total ovarian area was quantified using Image J software. The examined areas were determined in 3 ovarian sections from each ovary. The results were expressed as mean ± SEM of the positively labeled endothelial area.

### *Corpora lutea* vascular area

Lectin BS-1 (from *Bandeiraea simplicifolia*, 20 mg/ml), a proven constitutive endothelial cell marker^64,65^, was used to assess CL blood vessels. Ovarian sections were incubated with biotinylated lectin BS-1 overnight at 4 °C. After washing, the slides were incubated with avidin biotinylated horseradish peroxidase complex (Vectastain ABC system) for 30 min. Protein expression was revealed with diaminobenzidine (DAB) staining. Hematoxylin was used as a counterstain. The slides were then dehydrated before mounting (Canada Balsam Synthetic, Biopack, Argentina). Negative controls were obtained in the absence of lectin BS-1.

Vascular area (lectin BS-1-positive cells) was determined by thresholding the positive stained area and calculated by relativization to the total luteal area of the captured photograph using the Image J software (n = 3 animals/group, 16 ovarian sections/group).

### Hormone determinations by chemiluminescent immunoassay (CLIA)

For hormone determination, one group of animals received saline or ALLO 6 µM in both ovaries (n = 5 animals/group). Serum estrogen (E2), P4 and FSH concentrations were determined by CLIA using a Mindray CL-1200i analyzer (Mindray Laboratories, Shenzhen, China) and its specific reagents, with analytical sensitivity of ≤ 0.2 mIU/mL for FSH, ≤ 25 pg for E2, and ≤ 0.1 ng/mL for P4.

### Real-time PCR

Total RNA was obtained from ovarian fragments using the TRIZOL method according to the manufacturer’s instructions (n = 4 animals/group). Quantification of the RNA obtained was performed in a Jenway spectrophotometer at 260 nm. RNA integrity was verified by electrophoresis in a 1% agarose gel stained with SYBR Gold (Thermofisher Scientific Technologies, Buenos Aires, Argentina). Five micrograms of total RNA were retrotranscribed at 37 °C using random hexamer primers (Random Hexamers, Thermofisher) and murine leukemia virus retrotranscriptase enzyme (M-MLV, Thermofisher) in a reaction mixture with a final volume of 20 µl, according to previously published steps^21^.

Reactions were performed in a real-time Corbett Rotor Gene 6000 thermal cycler (Corbett Research Pty Ltd, Sydney, Australia), with a final volume of 20 µl. The reaction mixture consisted of 2 µL of 10 × PCR buffer, 1 µL of 50 mM MgCl2, 0.4 µL of 10 mM dNTPs, 1 µL of 20 × Eva Green (Biotium, Hayward, CA, USA), 0.25 µL of 5 U/µL Taq DNA Polymerase (Thermofisher), 0.1 µL of each primer (sense and antisense, detailed in Table 2) 2.5 mM, and 10 µL of cDNA diluted 1/20 in sterile RNAase-free H2O. PCR reactions were initiated with a 5-min incubation at 95 °C, followed by 40 cycles of 95 °C for 30 s, 60 °C for 30 s and 72 °C for 30 s.

**Table 2:** Primers used for quantitative polymerase chain reaction amplification.

Gene name	Forward primer (5’ to 3’)	Reverse primer (5’ to 3’)	GenBank accession N°	Fragment size (bp)
3β-HSD2	AGCAAGGACAGACATATAAGC	GTATCTCTGGGCAGGTATTTC	NM_017265.4	87
20α-HSD	CTTCCCATCGTCCAGAGTTG	GCAGAGATCCACTGTGTCAA	NM_138510.1	173
3α-HSOR	CGGAGTTACATTGATTATGGAGTT	GGTGTCAGAAGTCAGGAGAA	NM_138547.3	178
BAX	GCGATGAACTGGACAACAAC	CACACGGAAGAAGACCTCTC	NM_017059.2	86
BCL-2	TGGGATGCCTTTGTGGAACTA	AGAGACAGCCAGGAGAAATCAAAC	NM_016993.2	68
FAS	CCAGTAGCGTTCAGCGATG	GATGTCAGAGCAGTCATTCCT	NM_139194.2	192
FAS-L	GGCACAAGTCATTCTCTAC	TCGCTCTACTCTCATACATAA	NM_012908.1	82
Cyclin D1	AAGGGCTTCAATCTGTTCCTG	CCGGACTGCCTCCGTGCCT	NM_171992.4	42
S-16	TCCAAGGGTCCGCTGCAGTC	CATTCACCTTGATGAGCCCAT	NM_001169146.1	100

Melt curve analysis was used to verify that a single specific amplified product was generated. Real-time quantification was monitored by measuring the increase in fluorescence caused by the binding of EvaGreen dye to double-stranded DNA at the end of each amplification cycle. Relative expression was determined using the comparative quantitation method of normalized samples relative to the expression of a calibrator sample according to the manufacturer’s instructions^66^. Each PCR run included a non-template control and a sample without reverse transcriptase.

All measurements were performed in duplicate. Reaction conditions and amounts of cDNA added were calibrated so that the assay response was linear with respect to the amount of cDNA input for each pair of primers (Table 2). RNA samples were tested for DNA contamination by performing the respective PCR reactions without prior reverse transcription.

The reference gene used to normalize the results was ribosomal S16. For the selection of this gene, the expression stability of 3 candidate genes, β-actin, S16 and HPRT1 (hypoxanthine guanine phosphoribosyltransferase 1 enzyme), was estimated using the free software BestKeeper® version 1 [http://gene-quantification.com/bestkeeper.html]. S16 showed the lowest coefficient of variation between groups and the highest stability between treatments.

### Statistical analysis

The raw data obtained were analyzed using Graph Pad Prism V.8.0.1 for Windows (Graph Pad Software, La Jolla, CA, USA). Shapiro–Wilk normality test was performed before statistical analysis. Paired Student’s t-test was used to determine differences between the means of paired normally distributed groups. Unpaired Student’s t-test was used for hormone assays. Wilcoxon signed ranked test was used to determine differences between the means of paired non-normally distributed groups. The comparisons between the means of different groups were carried out using one-way ANOVA followed by Tukey’s post hoc. The Kruskal–Wallis test followed by Dunn’s multiple comparison method was used to analyze nonparametric results. Two-tailed values of *p* < 0.05 were considered significant.

## Results

### Experiment 1

#### Effect of ALLO 6 µM on ovarian steroidogenesis

**Figure 2:**
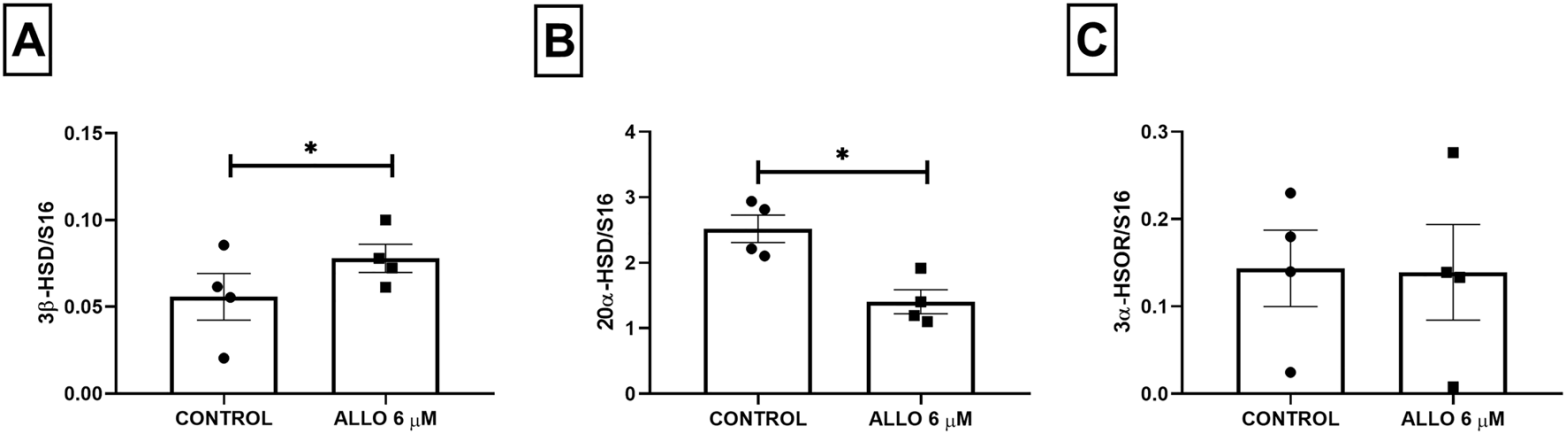
Gene expression of ovarian 3β-HSD2, 20α-HSD and 3α-HSOR after ALLO 6 µM intrabursal treatment. Results are expressed as mean ± SEM. Paired Student’s t test (*p < 0.05; n = 4 animals/experimental group).

Intrabursal administration of ALLO 6 µM induced a significant increase in 3β-hydroxysteroid dehydrogenase (3β-HSD2) (*p* = 0.0451) and a significant decrease in 20α-hydroxysteroid dehydrogenase (20α-HSD) enzyme gene expression (*p* = 0.0137). No significant changes in 3α-hydroxysteroid oxidoreductase (3α-HSOR) mRNA expression were observed (*p* = 0.9608, Fig. 2).

**Figure 3:**
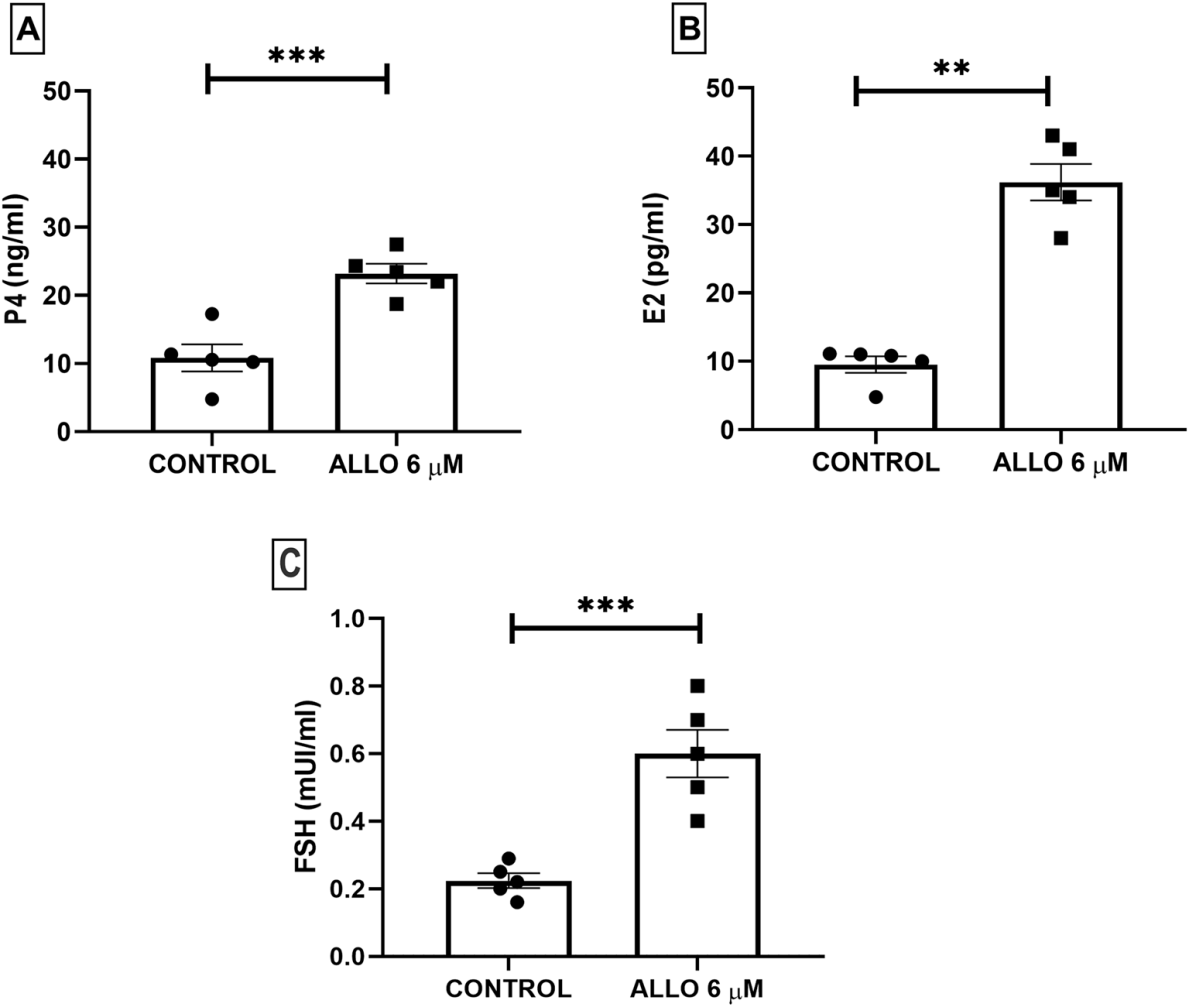
Effect of intrabursal administration of ALLO 6 µM on serum progesterone (**A**), estrogen (**B**) and FSH concentration. Results are expressed as mean ± SEM. (**A**) and (**C**) Unpaired Student’s t test, (**B**) Mann–Whitney test (**p < 0.01, ***p < 0.001; n = 5 animals/experimental group).

ALLO administration significantly increased P4 (*p* = 0.0010) and E2 (*p* = 0.0079) serum concentration (Fig. 3).

We also measured FSH serum levels. ALLO treatment induced a significant increase in this hormone (*p* = 0.0010, Fig. 3).

#### Effects of ALLO 6 μM on ovarian morphometry, weight and ovulation

**Figure 4:**
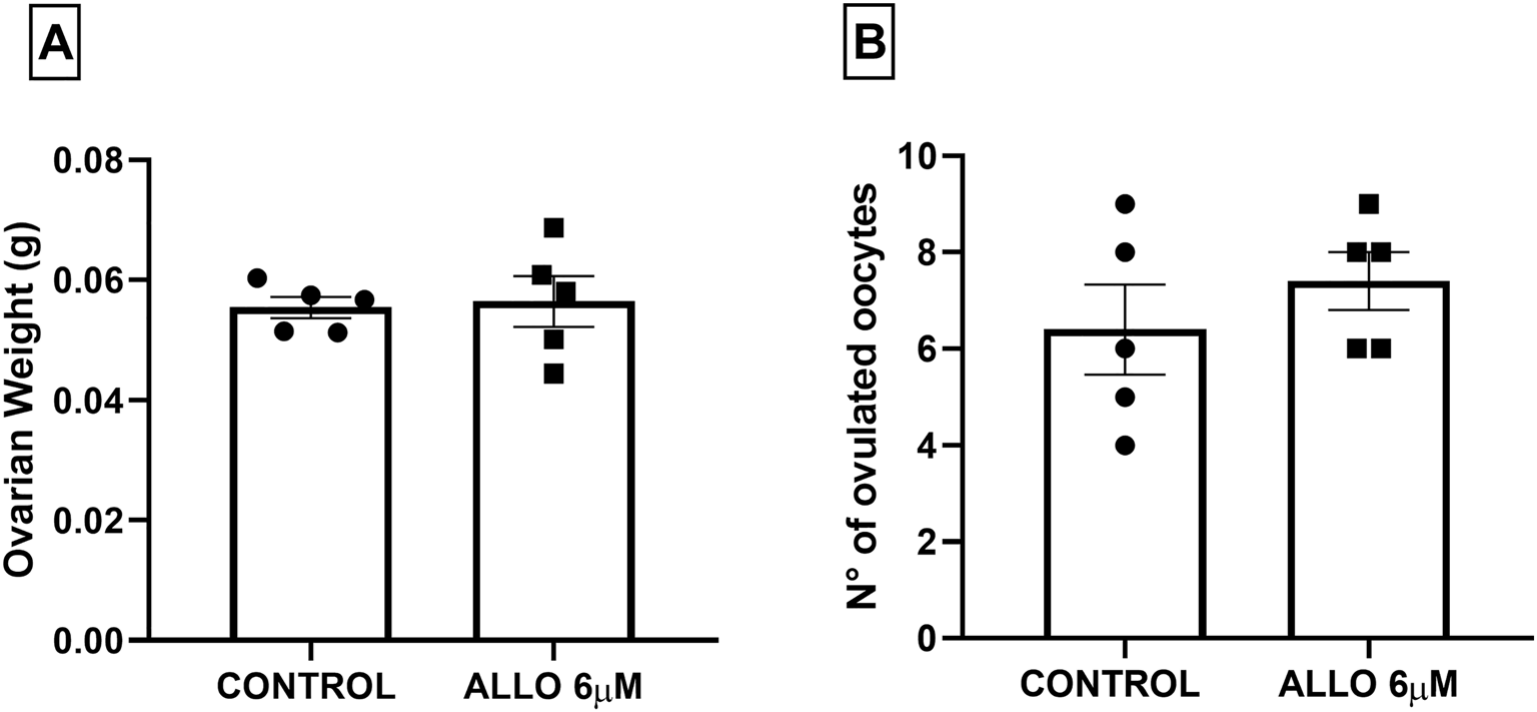
Ovarian weight in grams (**A**) and number of ovulated oocytes (**B**) in the ampullae after ALLO 6 µM intrabursal treatment. Results are expressed as mean ± SEM. Paired Student’s t test (ns > 0.05; n = 5 animals/experimental group).

ALLO intrabursal administration did not induce changes in ovarian weight (*p* = 0.7807, Fig. 4A) or in the number of ovulated oocytes (*p* = 0.5457; Fig. 4B).

**Figure 5:**
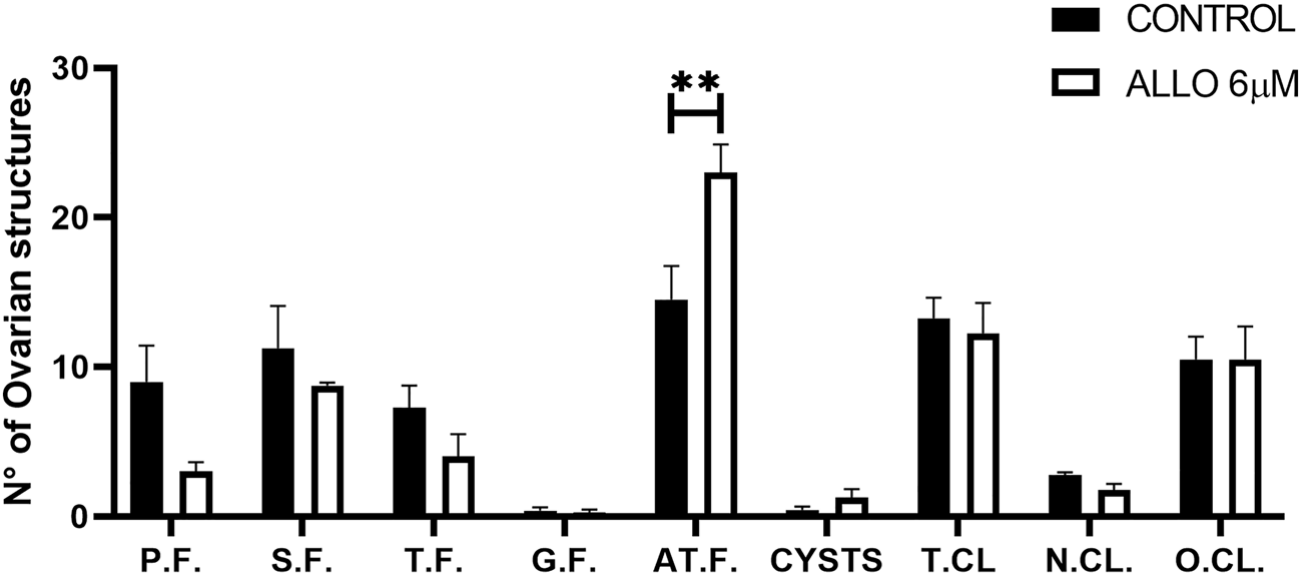
Effect of intrabursal administration of ALLO 6 µM on the number of structures per ovary. Primary follicles (P.F), secondary follicles (S.F), tertiary follicles (T.F), de Graaf follicles (G.F), atretic follicles (AT.F.), cysts, total number of corpora lutea (T.CL.), new corpora lutea (N.CL.) and old corpora lutea (O.CL.). Results are expressed as mean ± SEM. Two-way ANOVA with Bonferroni post hoc (n = 4 animals/experimental group, **p < 0.01).

ALLO administration resulted in a significant increase in the number of ATF (*p* = 0.0077). However, none of the other ovarian structures were significantly affected by the treatment (Fig. 5).

**Figure 6:**
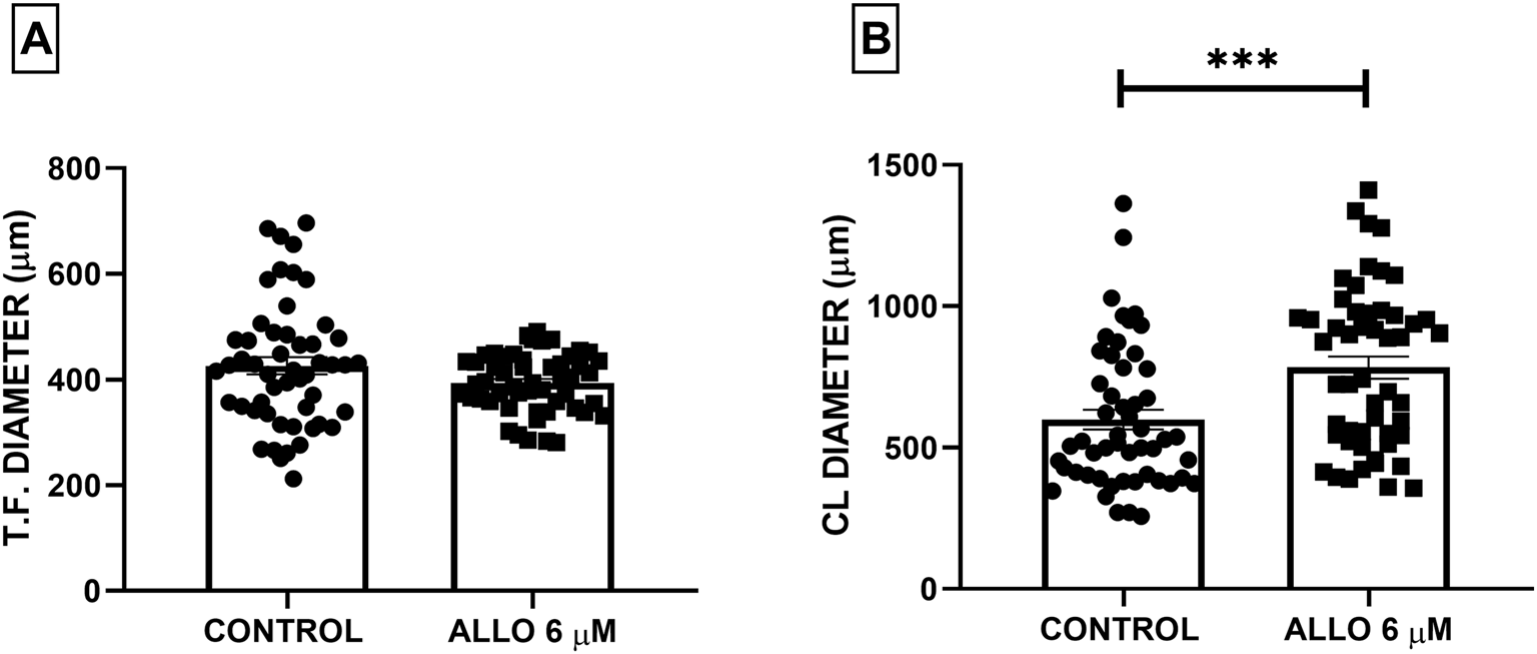
Effect of intrabursal administration of ALLO 6 µM on tertiary follicle (T.F.) and corpora lutea (CL) diameter. Results are expressed as mean ± SEM. Paired Student’s t-test and Wilcoxon signed ranked test (n = 5 animals/experimental group, ***p < 0.0001).

Regarding the diameter of the measured structures, ALLO 6 µM induced a significant increase (*p* < 0.0001) in the largest diameter of CL. The diameter of tertiary follicles was not affected (Fig. 6).

#### Ovarian BAX, BCL-2, FAS, FAS-L and cyclin D1 gene expression

**Figure 7:**
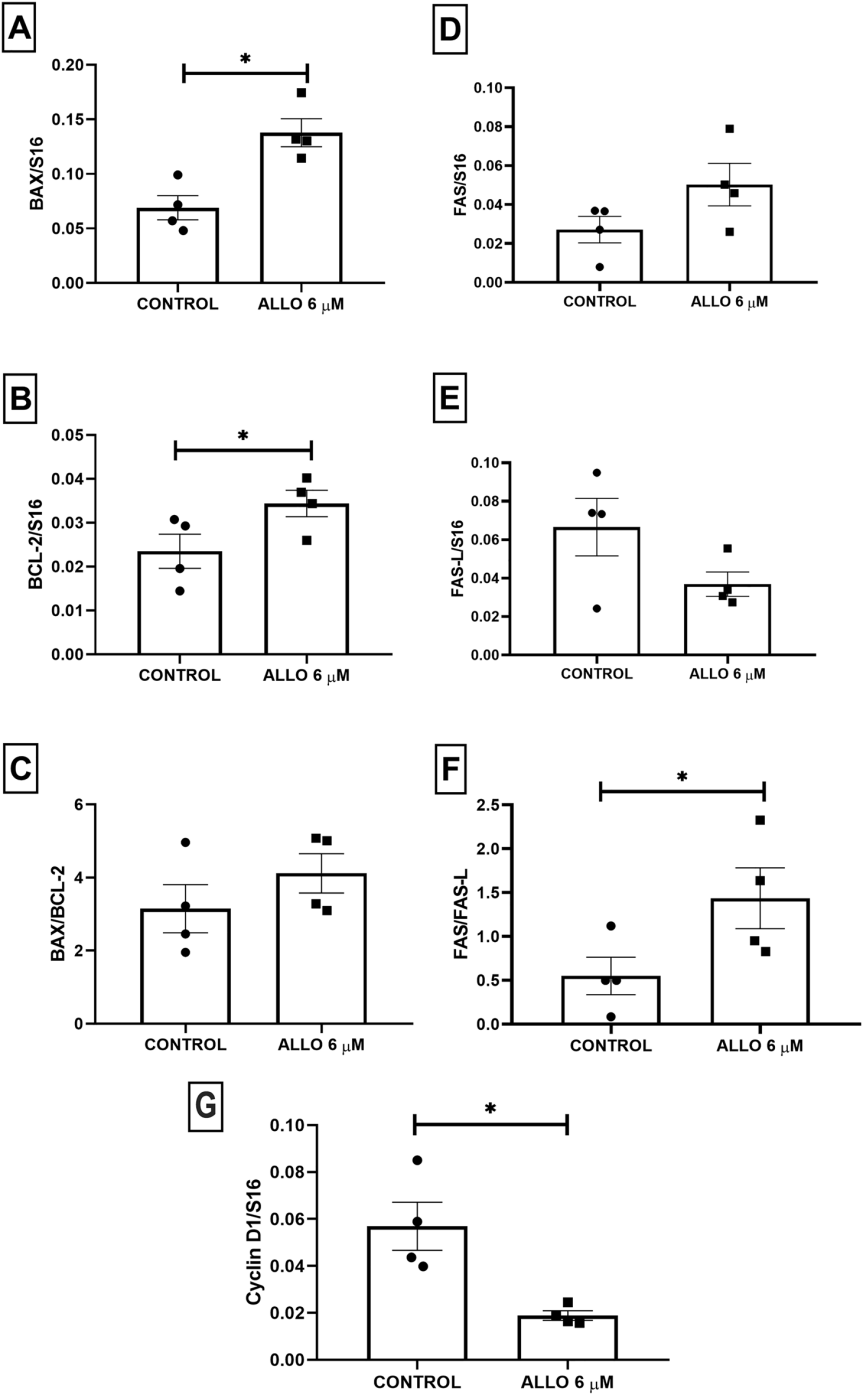
Effect of intrabursal administration of ALLO 6 µM on the expression of BAX, BCL-2, FAS, FAS-L and the BAX/BCL-2 and FAS/FAS-L ratio (**A**–**F**). (**G**) ALLO effect on cyclin D1 gene expression. Results are expressed as mean ± SEM. Paired Student’s t test (* < 0.05; n = 4 animals/experimental group).

ALLO 6 µM induced a significant increase in gene expression of both BAX (*p* = 0.0303) and BCL-2 (*p* = 0.0236), but without significant changes in the BAX/BCL-2 ratio (*p* = 0.2778, Fig. 7).

Regarding the extrinsic pathway of apoptosis, ALLO induced a significant increase in the FAS/FAS-L ratio (*p* = 0.0213, Fig. 7). ALLO administration did not significantly affect FAS (*p* = 0.0983) and FAS-L (*p* = 0.1714) gene expression.

Cyclin D1 gene expression was analyzed as a marker of ovarian cell proliferation. Intrabursal administration of 6 µM ALLO produced a significant decrease in this marker (*p* = 0.0465; Fig. 7).

#### Effect of ALLO 6 µM on follicular granulosa and theca cell proliferation and apoptosis

**Figure 8:**
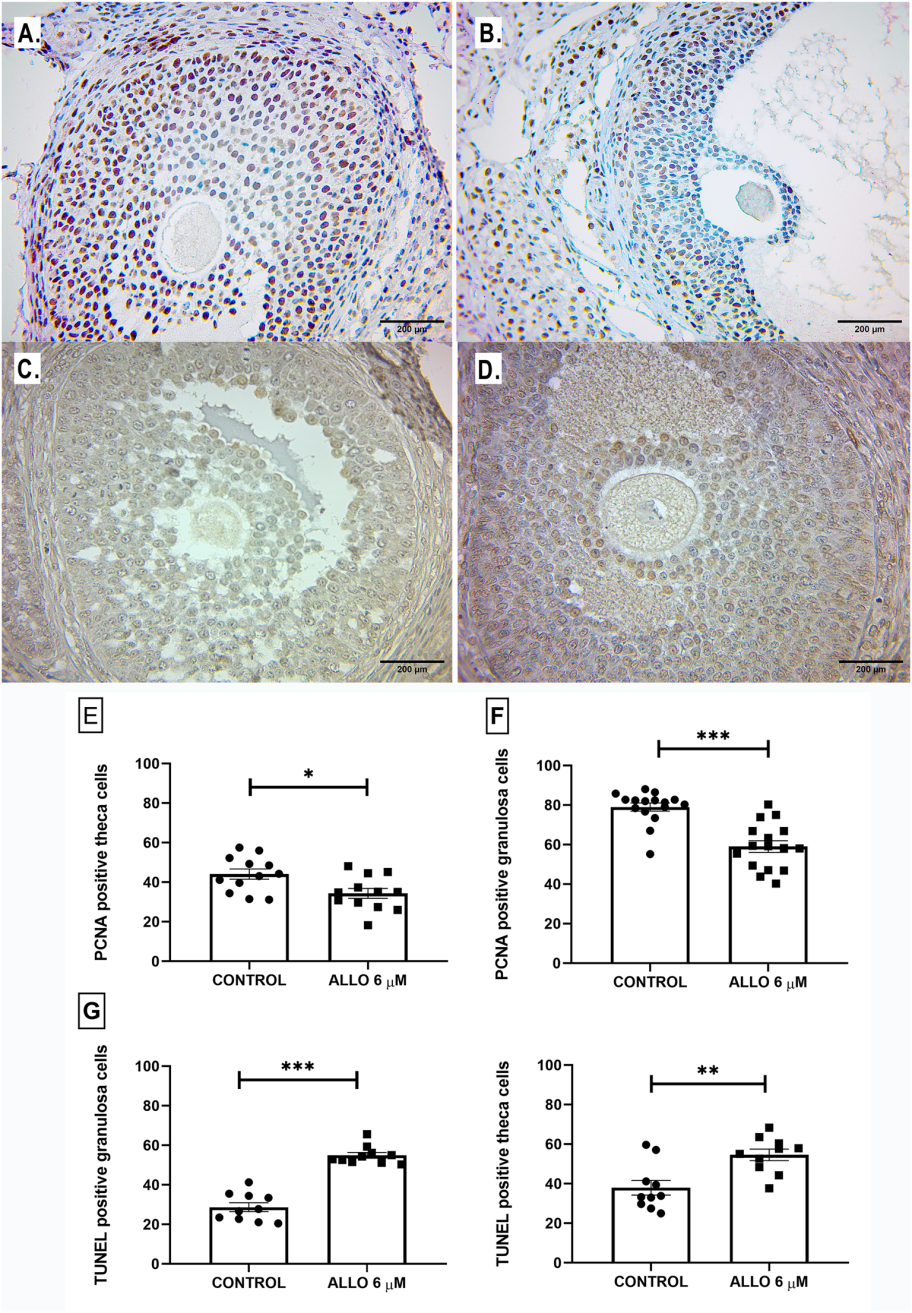
Upper panel: Representative microphotographs of tertiary follicles present in control (left panel) and ALLO 6 µM-treated (right panel), PCNA-labeled (**A** and **B**) and TUNEL-labeled (**C** and **D**) ovaries. Light microscopy, 100 x, bar = 200 µm. Lower panel: Proliferation (**E**, **F**) and apoptosis (**H**, **I**) rates of granulosa and theca cells of ovarian follicles. Number of positively immunolabeled cells per field. Results are expressed as mean ± SEM. Paired Student’s t test and Wilcoxon matched pairs signed rank test (granulosa PCNA) (*p < 0.05/**p < 0.01/***p < 0.001; PCNA: n = 16 sections/ovary for granulosa cells, n = 12 sections/ovary for theca cells, TUNEL: n = 10 sections/ovary for granulosa cells, n = 10 sections/ovary for theca cells, total of 5 ovaries/group).

Administration of ALLO 6 µM induced a significant decrease in the proliferation index of theca (*p* = 0.0312) and granulosa (*p* < 0.0003) cells, accompanied by a significant increase in the apoptotic index (theca: *p* = 0.0019 and granulosa: *p* < 0.0001; Fig. 8).

#### Effect of ALLO 6 µM on the process of luteal cell proliferation and apoptosis

**Figure 9:**
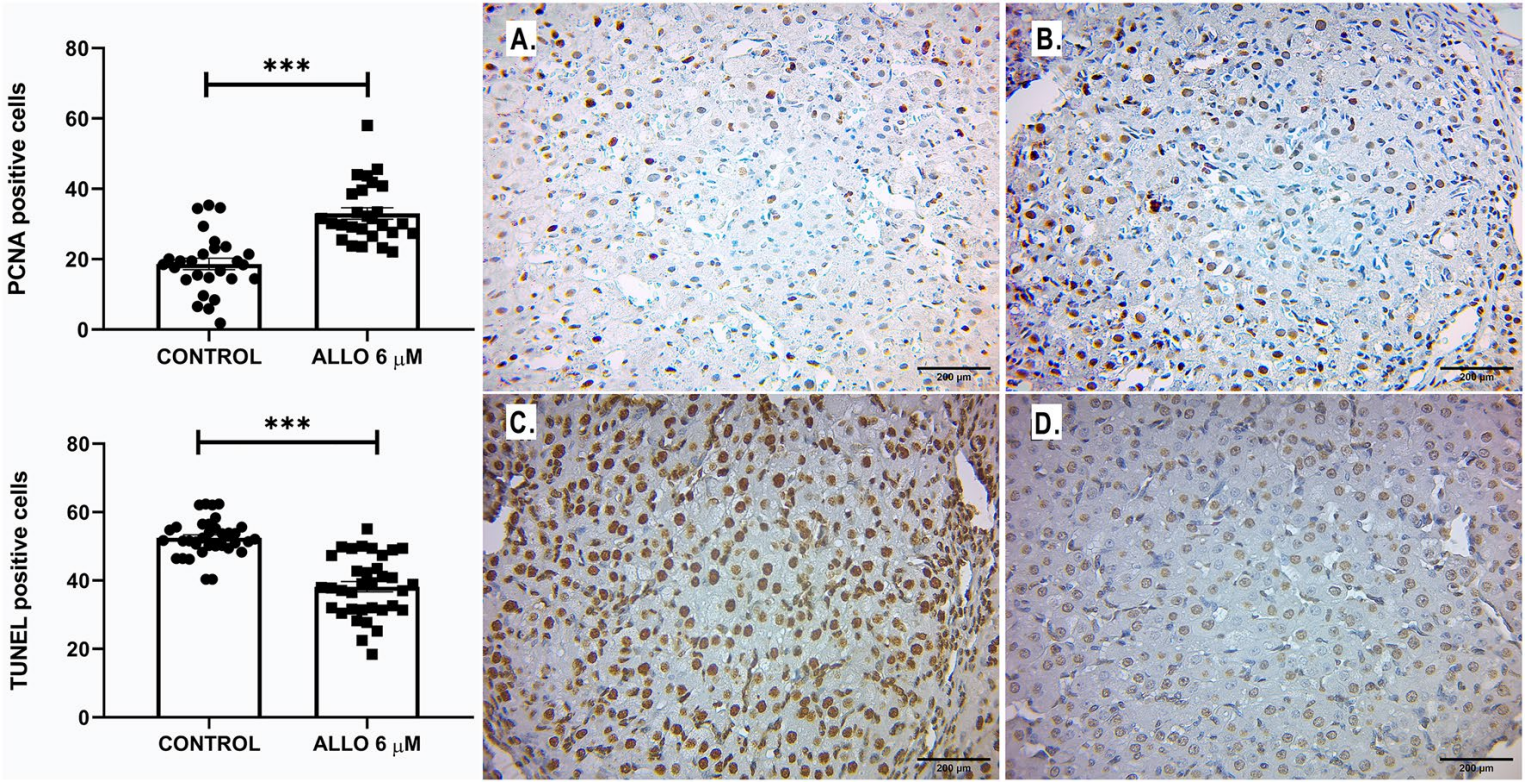
Rate of proliferation (upper panel) and apoptosis (lower panel) of luteal cells. Number of positively immunolabeled cells per field. Results are expressed as mean ± SEM. Wilcoxon matched pairs signed rank test (PCNA) and Paired Student’s t test (***p < 0.001). Representative microphotographs of CL present in control (left panel) and ALLO 6 µM-treated (right panel) ovaries, stained with PCNA (**A** and **B**) and TUNEL (**C** and **D**). Light microscopy, 100 x, bar = 200 µm (PCNA: n = 27 sections/ovary, TUNEL: n = 35 sections/ovary, total of 5 ovaries/group).

Paired intrabursal administration of 6 µM ALLO induced a significant increase in immunolabeling for PCNA in CL (*p* < 0.0001), accompanied by a significant decrease in TUNEL-positive cells (*p* < 0.0001; Fig. 9).

##### Ovarian angiogenesis

**Figure 10:**
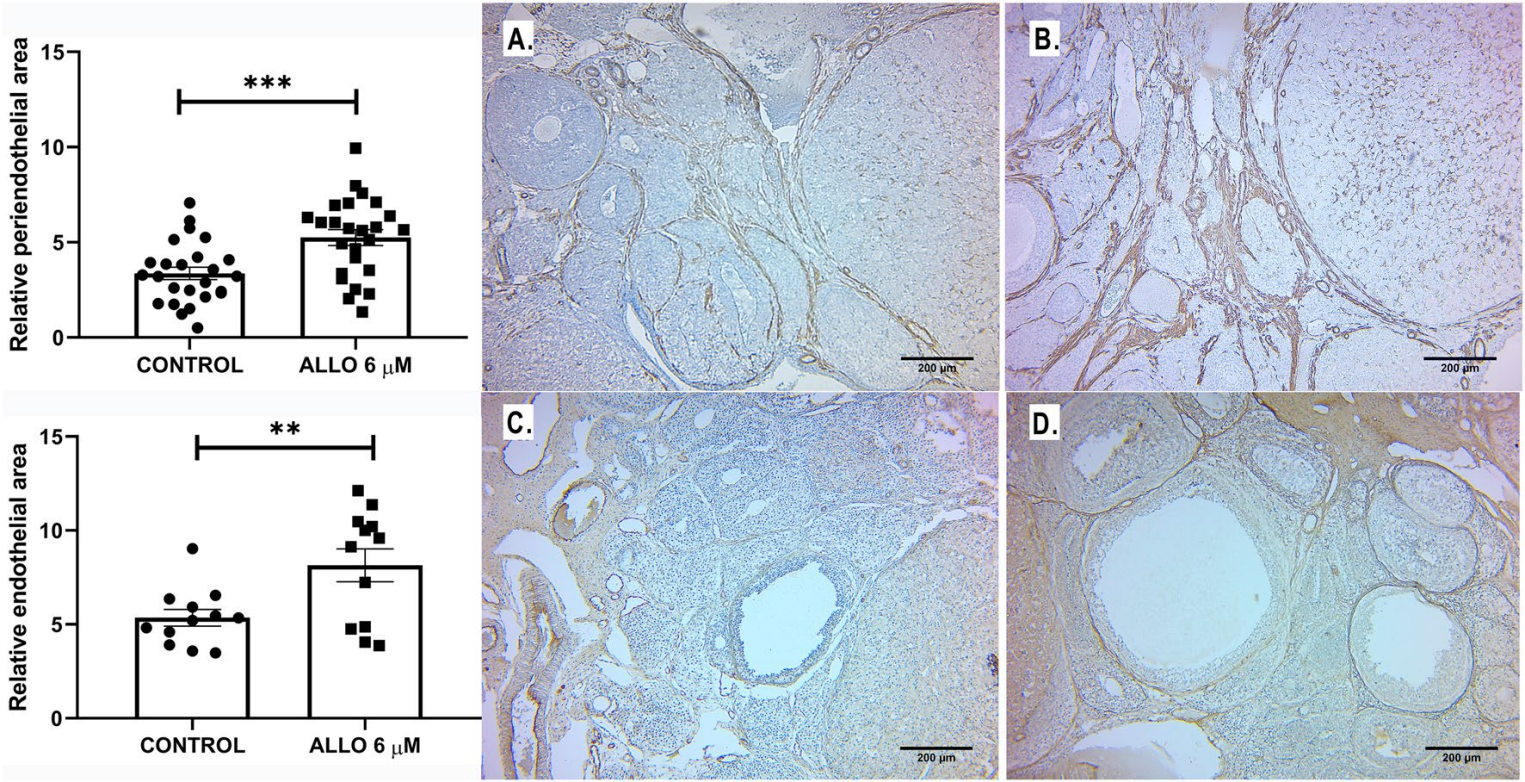
Relative periendothelial area labeled with α-actin (αA) and relative vascular area labeled with von Willebrand factor (VW). Results are expressed as mean ± SEM. Paired Student’s t test (*p < 0.05/**p < 0.01; n = 5 ovaries/group). Representative microphotographs of sections from control (**A** and **C**) and ALLO 6 µM-treated (**B** and **D**) ovaries, stained with α-actin (**A** and **B**) and Von Willebrand factor (**C** and **D**). Light microscopy, 400 x, bar = 200 µm.

The effect of ALLO 6 µM on vascular wall markers, α-smooth muscle actin and Von Willebrand factor was evaluated with the aim of analyzing angiogenesis. ALLO treatment significantly increased the percentage of relative vascular area for both markers (α-actin *p* = 0.0005; Von Willebrand *p* = 0.0058) compared to control (Fig. 10).

##### Corpora lutea angiogenesis

**Figure 11:**
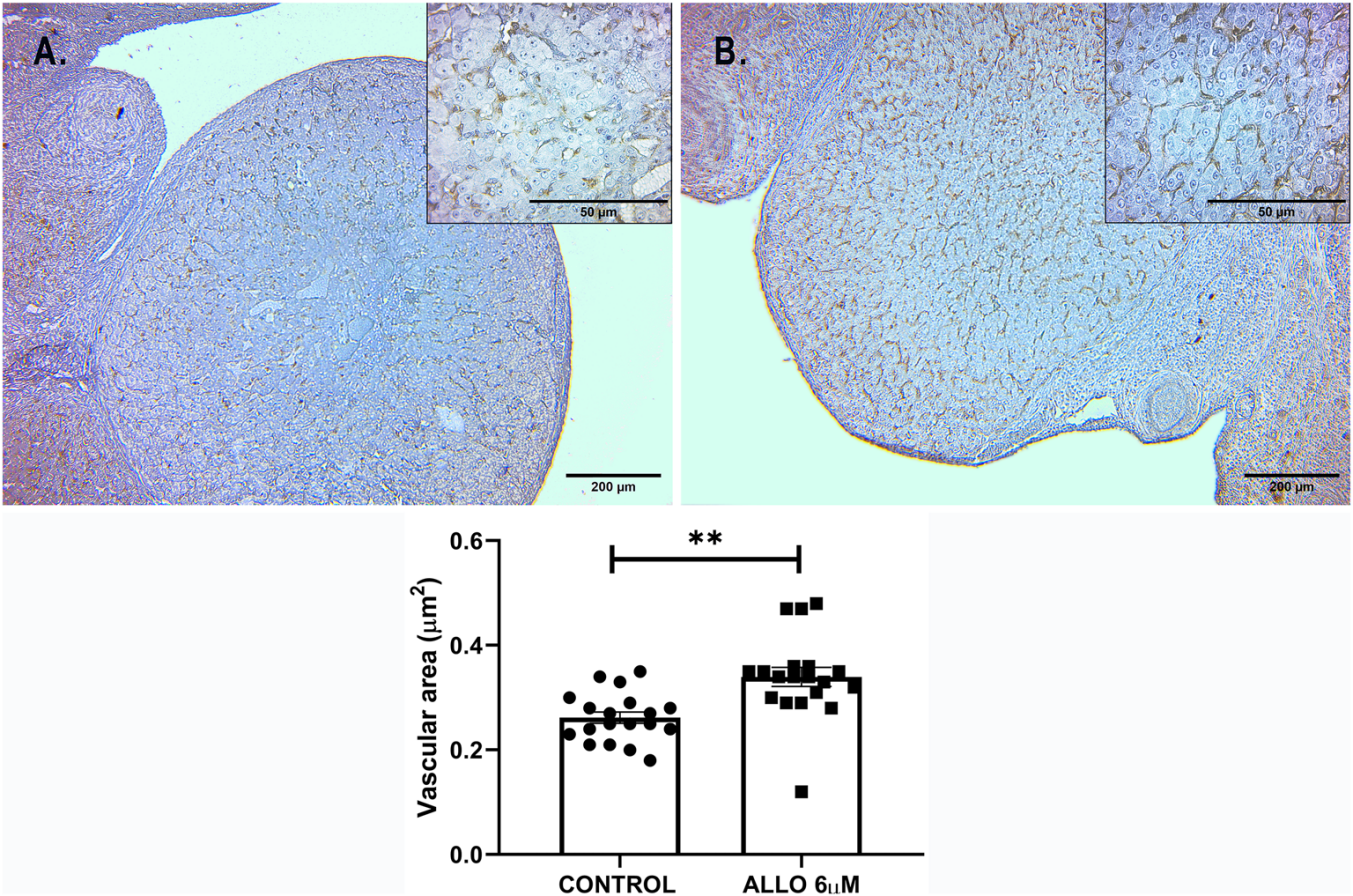
Upper panel: Representative microphotographs of sections from control (**A**) and ALLO 6 µM-treated (**B**) ovaries, stained with lectin BS-1. Light microscopy, 40 x, bar = 200 µm; inset 400 x, bar = 50 µm. Lower panel: Vascular area labeled with lectin BS-1. Results are expressed as mean ± SEM. Paired Student’s t test (*p < 0.05/**p < 0.01; n = 3 animals/group, 16 ovarian sections/group).

Lectin BS-1 staining significantly increased in CL after ALLO 6 µM treatment (Fig. 11).

##### Ovarian expression of the GABA_A_ Rα1-6 receptor

**Figure 12:**
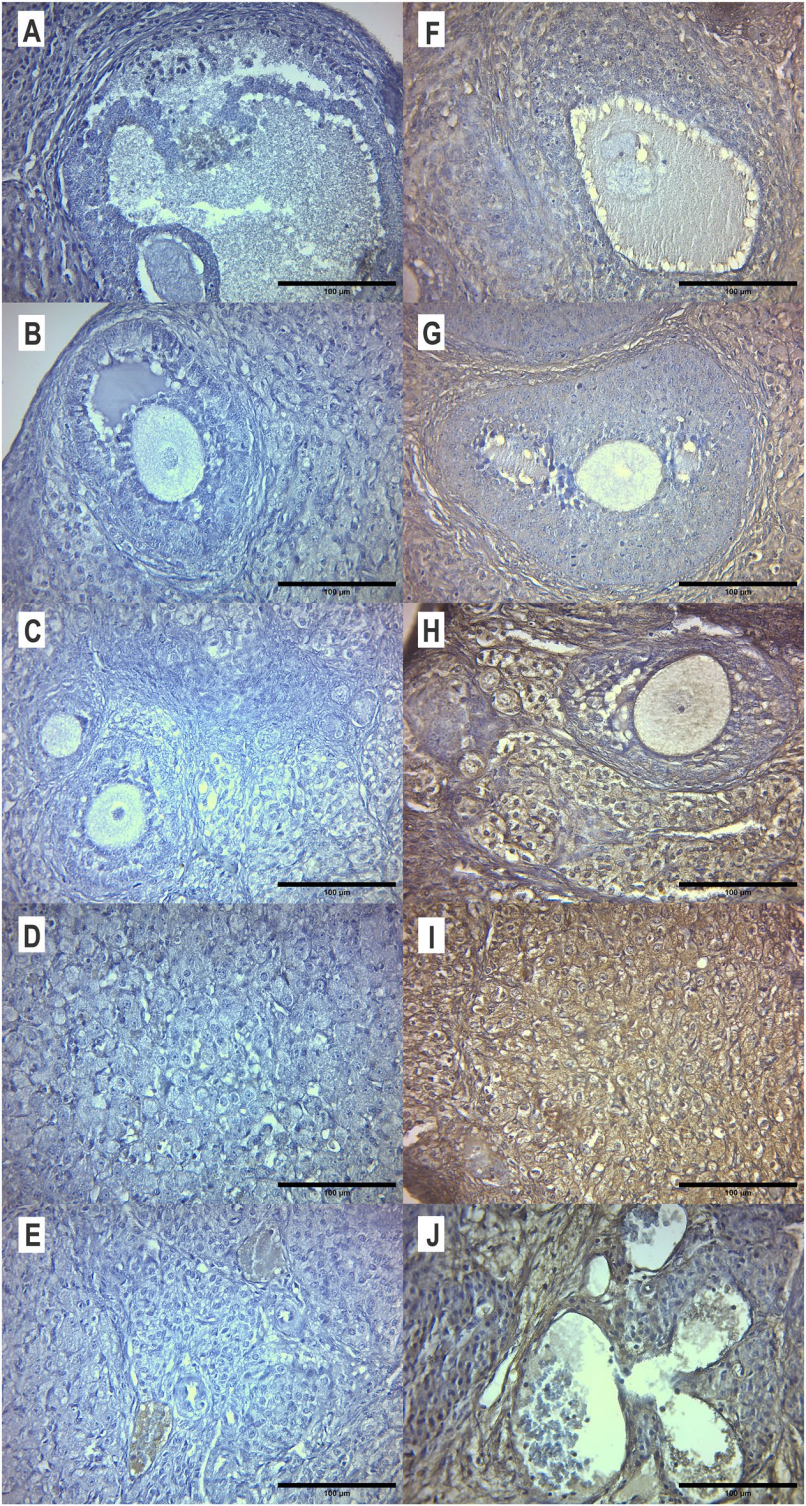
Effect of intrabursal ALLO 6 µM administration on the ovarian expression of the GABA_A_ receptor. Representative microphotographs of sections from control (left panel) and ALLO 6 µM-treated (right panel) ovaries, stained with anti GABA_A_ receptor Rα1-6. Atretic follicles (**A** and **F**), antral follicles (**B** and **G**), preantral follicles (**C** and **H**), *corpora lutea* section (**D** and **I**), stroma with blood vessels (**E** and **J**). Light microscopy, 400 x, bar = 100 µm.

The expression of the ovarian GABA_A_R staining increased after ALLO 6 µM treatment (Fig. 12). The positive mark was found in stroma, blood vessels, CL and the theca of healthy and atretic follicles.

### Experiment 2

#### Number of atretic follicles

**Figure 13:**
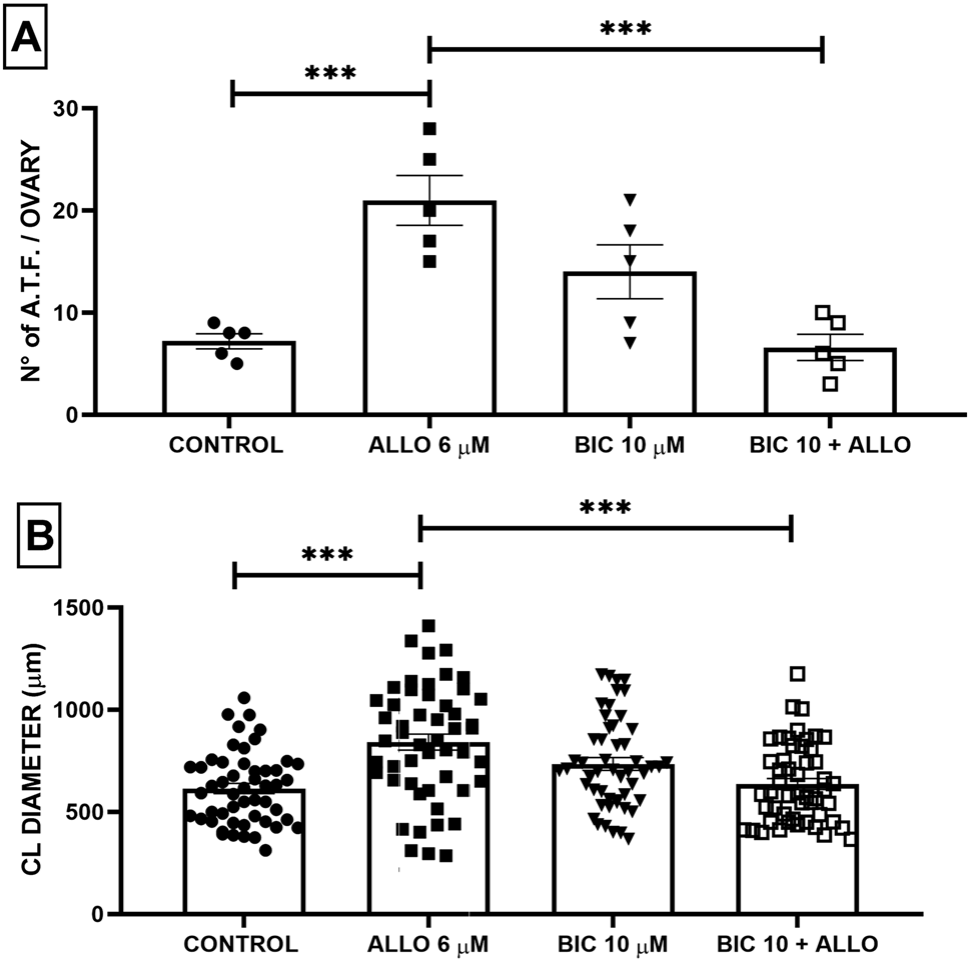
Effect of intrabursal ALLO 6 µM and BIC 10 µM administration on the number of atretic follicles (**A**) and on the diameter (µm) of CL (**B**). Results are expressed as mean ± SEM. (**A**) ANOVA I, post hoc Tukey; (**B**) Kruskal–Wallis with Dunn’s multiple comparison test (**p ≤ 0.01, ***p ≤ 0.0001; n = 5 animals/group, 51 CL/group).

BIC administration did not produce significant differences with respect to the control. The administration of BIC prior to ALLO prevented the increase in the number of ATF, showing a significant decrease compared to the administration of ALLO alone (F_(3, 16)_ = 12.10; *p* = 0.0002) (Fig. 13).

#### CL diameter

None of the BIC concentrations per se induced significant changes in CL diameter (*p* < 0.0001). When BIC was administered prior to ALLO, values returned to normal (Fig. 13).

## Discussion

Ovarian steroidogenesis and local steroid-mediated signaling is critical for proper ovarian function. An alteration in these processes may be the cause of various reproductive pathologies and infertility^67^. P4 biosynthesis and its metabolites are known as important regulators of the ovarian physiology. Considering that the concentration of P4 metabolites follows the same pattern as their precursor, and that the enzymes for its synthesis are found in ovarian tissue^24^, it is likely that derivatives are involved in local regulation. In this work, the effects of direct intrabursal ALLO administration were evaluated. It is important to note that the administration of the treatments was performed during the morning of the proestrus day, prior to the preovulatory LH peak, and at a time when ovarian P4 concentrations are low. The animals were sacrificed 24 h later, on the morning of estrus.

Plasma concentrations of E2 and P4 were measured to evaluate the endocrine response of the ovaries to the treatment. ALLO induced a significant increase in E2 concentration, detected on the morning of estrus. The peak of E2 during the normal estrous cycle of the rat typically occurs during the morning-afternoon of proestrus, with its plasma concentration decreasing towards estrus^68,69^. The increase in E2 during the follicular phase induces a positive feedback on GnRH neurons^70–73^, while a longer-lasting serum increase in E2 sensitizes the pituitary to GnRH action^74^. This coincides with the significant increase in plasma FSH produced by ALLO. As for the source of circulating E2, although an increase in the number of atretic follicles was observed, many were in early stages, so their granulosa cells (GCs) could still have been functional. Also, the increase in FSH could have stimulated the expression of follicular aromatase in preovulatory and non-ovulatory follicles, increasing secretion^75^. In addition, the CL of the rat retains its ability to secrete E2; hence, it could also have participated in this effect^76–78^.

ALLO significantly increased serum P4 concentration. The physiological peak of follicular P4 secretion occurs during the afternoon—night of proestrus, recovering basal values in the morning of estrus^68,69^. This peak is responsible for facilitating the preovulatory release of LH and occurs due to the action of FSH on the GCs of the preovulatory follicle^79^. Thus, an increase in P4 serum concentration could bring forward or magnify LH secretion^80^. It is known that an exogenous dose of subcutaneous P4 administered in the morning of proestrus produces a marked increase in ovarian periovulatory P4 secretion and sensitizes the tissue to the action of gonadotropins. In turn, FSH stimulation during estrus increases P4 synthesis by the GCs of the preovulatory follicle^79,81^. The results presented here suggest that the administration of ALLO during this period could have similar consequences, stimulating the release of P4 by CL and follicles. The increase in CL diameter produced by ALLO could also support this hypothesis. Another feasible hypothesis is that ALLO administration alters the inhibin-activin-follistatin system, necessary for the paracrine regulation of ovarian steroidogenesis and response to gonadotropins^82^, although more studies should be performed to confirm this.

There is evidence of the role of the GABAergic system in endocrine organs, where it acts as a modulator of hormone synthesis and release^43,83^. ALLO could be modulating the release of ovarian E2 and P4 through the GABA_A_R. Thus, the non-neural GABAergic system of these organs represents a possible therapeutic target for drugs that interact with GABA or its receptor. The changes in ovarian steroidogenesis induced by ALLO show effects that could destabilize the delicate endocrine balance. ALLO can alter hypothalamic regulation and induce pharmacological reprogramming of the CL. Pharmacological studies that include an in-depth analysis of systemic effects and implications are therefore of particular interest.

The intrabursal administration of ALLO 6 µM did not produce significant alterations in preovulatory follicles, thus allowing ovulation. Ovarian weight was also analyzed, and no significant differences were found for any of the treatments.

ALLO 6 µM treatment induced a significant increase in the number of atretic follicles interfering with follicular dynamics, affecting the selection and recruitment of dominant follicles for the following cycle. No significant changes in the diameter of tertiary follicles were observed. As for the CLs present in the treated ovaries, their number was not greater than that of the controls, but their diameter was significantly higher. During estrus, CLs from previous cycles are in different stages of luteolysis, showing areas of cell degeneration, fibrosis, and a reduction in size. At this stage, the newly formed CLs begin to increase in size^84^. As no changes in the number of ovulated oocytes were recorded, the increase in CL diameter could be interpreted as a delay in luteolysis of CL corresponding to previous cycles.

Pharmacological “reprogramming” of rodent CLs that have already embarked on the path of luteolysis is possible^85^. A postpartum subcutaneous injection of P4 in rats can inhibit luteal cell apoptosis^86^. Intrabursal administration of P4 to pregnant rats after activation of luteolysis by LH blocks induction of 20α-HSD activity, increases serum P4 concentrations and decreases ovarian PGF2α concentrations^87^. Thus, ALLO could interact with CL from previous cycles, recovering them from luteolysis. This finding could be useful for the treatment of reproductive conditions that involve deficiencies in CL functionality, or that are caused by early regression of the CL.

The expression of ovarian steroidogenic enzymes involved in P4 synthesis and metabolism was analyzed by real-time PCR. Treatment with ALLO induced an increase in the enzyme 3β-HSD2, which synthesizes P4, accompanied by a decrease in 20α-HSD, which metabolizes it to a derivative lacking progestational activity^88,89^. These findings are compatible with the increase in plasma P4 produced by ALLO and a possible luteotrophic effect. There were no significant changes in 3α-HSOR gene expression. ALLO does not seem to regulate mRNA expression of the enzyme responsible for the final step in its synthesis, unlike what happens with P4 in the rat^90,91^.

The expression of pro- and anti-apoptotic ovarian markers varies cyclically and between ovarian structures, determining their fate. ALLO treatment induced an imbalance in ovarian pro- and anti-apoptotic markers, with a significant effect on the FAS/FAS-L ratio, showing an anti-apoptotic effect that could correlate with the inhibition of luteolysis. During luteolysis, prolactin activates CL apoptosis via the FAS/FAS-L system^92^, and ALLO administration may override this effect. Cyclin D1 is a marker of cell proliferation that shows higher expression in non-luteinised theca and granulosa cells^93^. Its gene expression was significantly reduced after ALLO treatment, supporting previous findings of ALLO-mediated follicular atresia.

It is important to highlight that the ovarian parenchyma comprises two histologically and biochemically different structures that undergo cyclic changes. The mRNA expression results presented correspond to the processing of the whole ovary, making it difficult to determine which structure was affected. For the correct interpretation of results, it was necessary to complement them with other techniques to differentiate the effects on follicles and CL. Hence, a TUNEL technique and PCNA immunohistochemistry were performed. There was an increase in apoptosis of GCs and follicular theca cells, accompanied by a significant decrease in PCNA labeling, in concordance with the histological count. In the case of CLs, an opposite picture was observed, with a decrease in TUNEL labeling and an increase in PCNA. This result supports the luteotrophic effect of ALLO.

The process of apoptosis in the ovary is closely linked to variations in hormone secretion, driven by the regulation of the estrous cycle^94^. The increased follicular atresia found could be related to the hormonal imbalance caused by ALLO. Several studies^95–99^ have shown that the follicular fluid of atretic follicles contains altered levels of E2 and P4. However, elevated circulating concentrations of FSH, E2 and P4 could generate an ovarian trophic microenvironment, as they all possess anti-apoptotic effects on GC^63,100–105^. Thus, the ALLO stimulus on non-dominant follicles is decisive, since it occurs in an environment where other stimuli that inhibit follicular atresia are abundant. It is also possible that GABA_A_R activation induces an efflux of cellular chloride ions. In non-neural cells, chloride ions flow in both directions, according to the cellular electrochemical gradient, regulating osmotic tension^106^. Changes in intracellular ion concentration and cell volume could induce apoptosis^107^.

The development of the ovarian structures, as well as their function, depends on correct blood flow that allows the arrival of endocrine regulatory factors and the transport of the hormones produced. It has been demonstrated that the expression of angiogenic factors in the ovary is hormone-dependent; both the follicles and the CLs produce angiogenic factors^108,109^, which are then regulated by endocrine and paracrine factors. An increase in follicular vascularization selects dominant follicles, whereas its reduction is one of the first signs of atresia^110,111^. In the mature CL, approximately 50% of its cells correspond to endothelial cells^112^, and their blood flow correlates with P4 secretion^113^. Conversely, the decrease in CL blood flow is associated with luteolysis^109,114^. ALLO induced a significant increase in ovarian vascular and periendothelial area, immunolabeled with Von Willebrand and α-actin, respectively. This effect could be explained by the interaction between ALLO and the endothelial GABAergic system, where GABA_A_R activation induces an increase in cell proliferation and GABA synthesis^115–117^. ALLO could also modulate pro-angiogenic factors. We showed an increase in lectin BS-1 staining in the CL of ALLO treated ovaries; this finding could indicate increased blood flow to persistent CLs that have increased in diameter and functionality, thus increasing P4 secretion.

Functional studies determine the action of the ligand on a receptor and aid in the pharmacological characterization of the mechanism of action. In this case, to assess whether the action of ALLO in the ovarian parenchyma is GABA_A_R-dependent, a functional experiment was performed with BIC, a competitive antagonist of the receptor. This is the assay of choice to evaluate the interaction of agonists with GABA_A_R^44^.

GABA not only has functions on the central and peripheral nervous systems but is also present in a wide variety of peripheral tissues such as the intestine, stomach, gonads, and others^118^. Although the presence of GABA_A_R and a non-neural GABAergic circuit in the ovary is known, there are no studies that delve into its reproductive functionality. All the available studies correspond to modulation of the CNS and PNS^7,9,119,120^. The study of the effects of GABA_A_R system in the ovary is of particular interest due to the possible relationship with certain pathologies, such as polycystic ovary syndrome (PCOS) and ovarian cancer, as well as possible undesirable effects of the systemic application of ALLO treatments. In a previous study, ALLO administration to the superior mesenteric ganglion significantly increased GABA_A_ receptor protein expression in the ovary. In this work, ALLO intrabursal administration had a similar effect, increasing GABA_A_ receptor staining in stroma, blood vessels, CL and the theca of healthy and atretic follicles. This effect might increase ovarian GABAergic effects and thus be responsible for some of ALLO actions.

When BIC 10 µM was administered prior to ALLO, a return to control-like values of the number of atretic follicles and CL diameter was observed. These results indicate that the action of ALLO on these structures is mediated through GABA_A_R. There is a differential effect between structures: apoptosis in follicles is promoted, while luteolysis is delayed. These opposing effects could be explained by considering the plasticity of the receptor present in the different structures.

In the CNS, GABA_A_R shows plasticity throughout the estrous cycle, so that its expression and conformation seem to be linked to the circulating concentration of P4 and ALLO^121–126^. The physiological response of GABA_A_R depends on the subunit composition of the receptor, which determines its functional properties^127,128^. For example, GABA_A_Rs containing the ɗ subunit are more sensitive to the action of neuroactive steroids such as ALLO^8,129^. Finally, GABA_A_R distribution in the CNS is region-specific^130,131^. GABA_A_R activation may thus vary between receptor composition and location. One might expect that these same changes could occur in non-neural tissues. Fujii and Mellon’s work^42^ corroborates this theory; they report changes in the subunit composition of the uterine GABA_A_R during pregnancy and postpartum in the rat, with the subunits exhibiting differing sensitivity to ALLO. Thus, it would also be expected that the effects assessed in this work would be different in the different stages of the rat’s estrous cycle.

In conclusion, the administration of intrabursal ALLO 6 µM alters several processes of the ovarian morpho-physiology of the female rat, such as luteal regression, follicular atresia, angiogenesis and ovarian steroidogenesis, which could be related to oocyte quality and fertility. ALLO 6 µM exerts a pro-apoptotic effect on growing follicles, which is proliferative in CL and pro-angiogenic in the ovary. It also alters steroidogenesis and hormonal balance at the estrous stage. Some ALLO effects occurred through the GABA_A_R, as they were be inhibited with BIC.

Dysregulations of the processes of apoptosis^132^, steroidogenesis^25,133,134^ and angiogenesis^135^ are related to ovarian pathologies such as PCOS and cancer. The study of the ovarian effects of GABA_A_R modulation by neuroactive steroids is of particular importance since they modulate key aspects of reproductive physiology.

## Data Availability

The datasets generated during and/or analyzed during the current study are available from the corresponding author on reasonable request.
